# Optimization with artificial intelligence of the machinability of Hardox steel, which is exposed to different processes

**DOI:** 10.1038/s41598-023-40710-8

**Published:** 2023-08-29

**Authors:** Mehmet Altuğ, Hasan Söyler

**Affiliations:** 1 Malatya Organized Industrial Zone (OIZ) Vocational High School, Inonu University, Malatya, Turkey; 2 Faculty of Economics and Administrative Sciences Econometrics Department, Inonu University, Malatya, Turkey

**Keywords:** Mechanical engineering, Statistics

## Abstract

In this study, different process types were processed on Hardox 400 steel. These processes were carried out with five different samples as heat treatment, cold forging, plasma welding, mig-mag welding and commercial sample. The aim here is to determine the changes in properties such as microstructure, microhardness and conductivity that occur in the structure of hardox 400 steel when exposed to different processes. Then, the samples affected by these changes were processed in WEDM with the box-behnken experimental design. Ra, Kerf, MRR and WWR results were analyzed in Minitab 21 program. In the continuation of the study, using these data, a prediction models were created for Ra, Kerf, MRR and WWR with Deep Learning (DL) and Extreme Learning Machine (ELM). Anaconda program Python 3.9 version was used as a program in the optimization study. In addition, a linear regression models are presented to comparison the results. According to the results the lowest Ra values were obtained in heat-treated, cold forged, master sample, plasma welded and mig-mag welded processes, respectively. The best Ra (surface roughness) value of 1.92 µm was obtained in the heat treated sample and in the experiment with a time off of 250 µs. Model F value in ANOVA analysis for Ra is 86.04. Model for Ra r^2^ value was obtained as 0.9534. The lowest kerf values were obtained in heat-treated, cold forged, master sample, plasma welded and mig-mag welded processes, respectively. The best kerf value of 200 µ was obtained in the heat treated sample and in the experiment with a time off of 200 µs. Model F value in ANOVA analysis for Kerf is 90.21. Model for Kerf r^2^ value was obtained as 0.9555. Contrary to Ra and Kerf, it is desirable to have high MRR values. On average, the highest MRR values were obtained in mig-mag welded, plasma welded, cold forged, master sample and heat-treated processes, respectively. The best mrr value of 200 g min^−1^ was obtained in the mig-mag welded sample and in the experiment with a time off of 300 µs. Model for MRR r^2^ value was obtained as 0.9563. The lowest WWR values were obtained in heat-treated, cold forged, master sample, plasma welded and mig-mag welded processes, respectively. The best wwr value of 0.098 g was obtained in the heat treated sample and in the experiment with a time off of 200 µs. Model F value in ANOVA analysis for WWR is 92.12. Model for wwr r^2^ value was obtained as 0.09561. In the analysis made with artificial intelligence systems; The best test MSE value for Ra was obtained as 0.012 in DL and the r squared value 0.9274. The best test MSE value for kerf was obtained as 248.28 in ELM and r squared value 0.8676. The best MSE value for MRR was obtained as 0.000101 in DL and the r squared value 0.9444. The best MSE value for WWR was obtained as 0.000037 in DL and the r squared value 0.9184. As a result, it was concluded that different optimization methods can be applied according to different outputs (Ra, Kerf, MRR, WWR). It also shows that artificial intelligence-based optimization methods give successful estimation results about Ra, Kerf, MRR, WWR values. According to these results, ideal DL and ELM models have been presented for future studies.

## Introduction

WEDM is an unconventional manufacturing process commonly used to process conductive high-strength materials. WEDM is adept at producing complex and complex shapes^1–3^. The very important machining responses of the process are the ideal metal removal rate, ideal the roughness of the finished surfaces, and the effective cutting width, which is the notch. Kara examined the optimum results in the finishing milling of Hardox 400 using the Taguchi method in his study^4^. Kerf is specified as the cutting width in WEDM activities. This depends on cutting parameters such as gap time between two pulses, wire feed, servo voltage, dielectric fluid pressure and wire tension^1–3^. Manoj et al.^1^ investigated changes in cutting speed, surface roughness, recast layer and microhardness in wedm using genetic algorithm. Using Taguchi experimental design method, it was found that factors for instance discharge current, pulse time and dielectric rate and their interactions have a significant effect on rough cutting operations in order to maximize material removal rate and minimize Ra and cutting width. Nas et al.^5^ investigated the effects of machining parameters on the experimental and statistical results using the electric discharge method in the machining of AISI D2 cold work tool steel. Nas and Kara investigated machinability tests on a corrosion resistant superalloy subjected to shallow (SCT) and deep cryogenic machining (DCT) with Electric erosion machining (EDM) and the effect of cryogenic treatment types applied to the material on EDM machining performance^6^. Bayraktar and Kara^7^ investigated the effect of deep cryogenic treatment on surface roughness parameters of Sleipner cold work tool steel using PVD coated carbide tools.

Ra estimation have been divided into three classes^8^, Methods based on machining process theory. The surface morphology is modelled and simulated by the analytical model, and then the Ra is calculated from the simulated surface morphology. Methods based on interrupt signals. Inferences are made on acoustic emission, vibration, and shear force signals and the most relevant feature quantities are determined to examine existing artificial intelligence based Ra methods. With algorithms, the mathematical structure between cutting parameters or cutting signals and Ra is established and Ra is estimated^8–10^.

Artificial intelligence (AI)-based approaches are suitable for existing data-driven production environments as they can integrate with production systems. For Ra prediction methods with artificial intelligence, Zhang et al. Built-in parallel convolution module to extract multidimensional feature information from Ra images. Then, Ra was evaluated with the deep learning model in the light of the extracted information^11^. Li et al.^12^ suggested an improved fireworks algorithm to monitor grinding Ra with force signals. Guo et al.^13^ suggested Ra prediction with features extracted from vibration, grinding force and acoustic emission signals. Patel and Gandhi^14^ studied the parameters affecting Ra in turning D2 steel. Tian et al.^15^ In their studies, Ra was estimated with fuzzy learning system together with process parameters and different signal properties. García Plaza et al.^16^ investigated how Ra would be affected by vibrational signals. Nguyen et al. developed a model for monitoring grinding wheel wear. It showed that the model could accurately predict Ra on the grinding surface with 98% confidence^17^.

By using AI and ML methods, fuzzy logic, artificial neural network, genetic algorithm, ANFIS and other methods have been made easier to solve engineering problems. Additionally, swarm intelligence optimization algorithms were used to estimate Ra based on tool wear^10,18^. With DL network structures, it is provided to improve tracking accuracy and learn multi-scale features^19,20^. Guleria et al.^21^ created a Ra model with an extreme learning machine (ELM) using effective features selected from the vibration signal as input. Erkan et al. a GFRP composite material was milled to experimentally minimise the damages on the machined surfaces, using two, three and four flute end mills at different combinations of cutting parameters. Also, study, Artificial Neural Network (ANN) models with five learning algorithms were used in predicting the damage factor to reduce number of expensive and time-consuming experiments^22^. Pimenov et al.^23^ adopted random forest to predict Ra according to tool wear and spindle power, but the prediction accuracy is not effective. Zhao et al.^24^ suggested a tuning method for Ra stabilization via the digital twin concept. Li et al.^25^ proposed a meta-learning model to predict tool wear. This model facilitated the interpretability of DL algorithms. Zhou et al.^26^ In order to optimize the cutting parameters, a Ra prediction model was established with an artificial neural network. Yan et al.^27^ introduces residual network with two-dimensional time–frequency domain signal for tool wear monitoring. Dedeakayoğulları et al.^28^ generated a Ra model through optimized neural network and cutting parameters.

**Figure 1:**
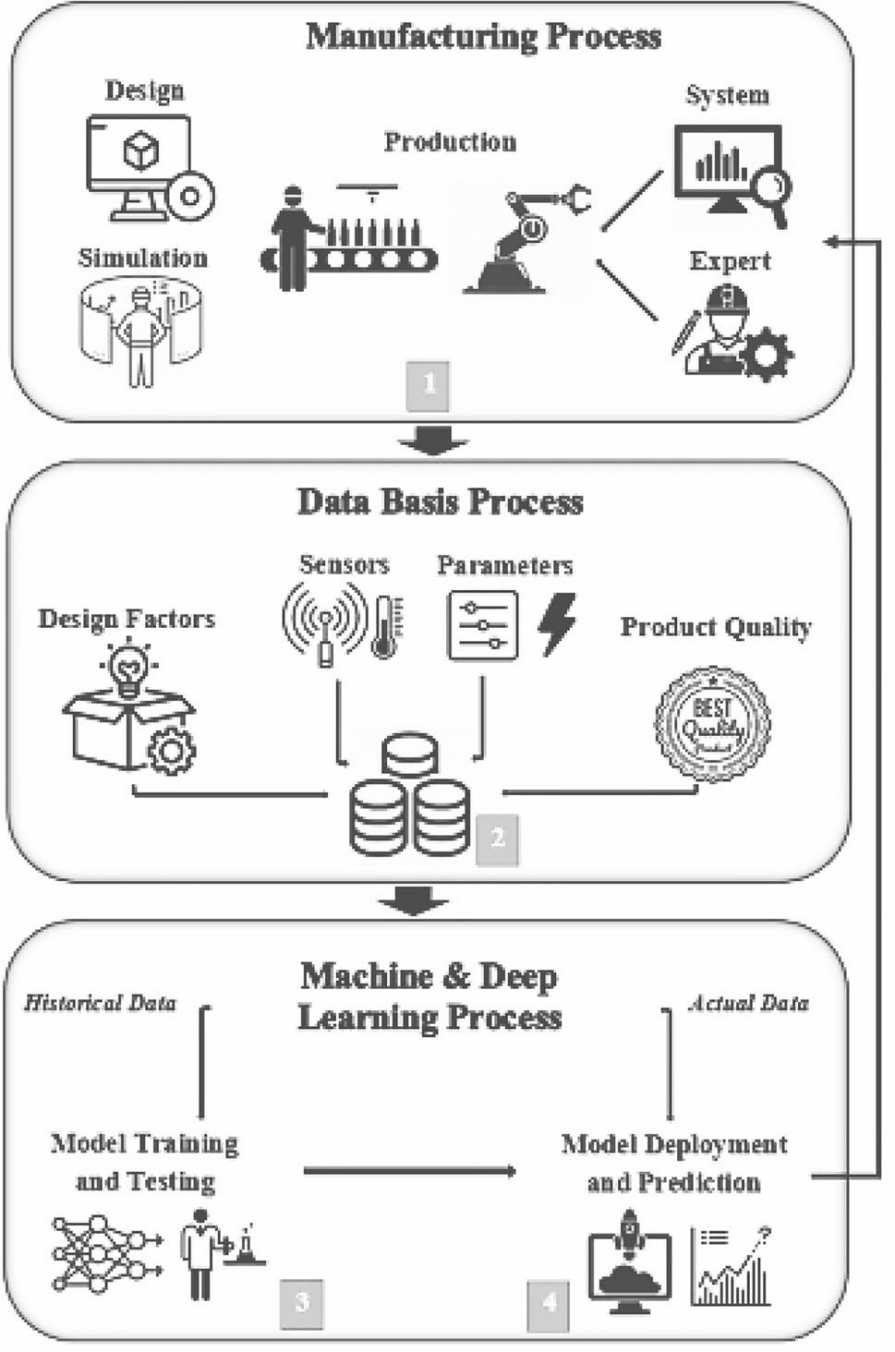
Predictive quality approach^29^.

Machine learning, deep learning, and other prediction solutions rely on data from the manufacturing process. The predictive view for quality allows product quality to be evaluated based on process data by removing repetitive template from the data and linking them to quality measures. In this process, evaluations form the basis for making decisions about quality improvement measures, such as adjusting parameters to avoid losses. The general approach to quality forecasting has four stages: formulating a production process and a quality objective, selecting and collecting process and quality data, running a learning model, and using a scoring model as the basis for decision making (Fig. 1). In this sense, estimated quality essentially involves supervised machine learning techniques^29^.

Researchers have effectively used methods such as ANN, ML and DL to increase efficiency. Ziletti et al.^30^ proposed an ML-based approach towards not automatically classifying their work. Researchers used a machine learning prediction method for proposing to stimulate the design of medical parts Ti alloys with low modul^31^. Cardoso Silva et al.^32^ specified that machine learning approximation was laid out as systems to help machine learning systems work, provide appropriate resources, and make decisions. Zhang et al.^33^ contemplated alloys with superior properties by iteratively designing composition by means of Bayesian optimization using the ML strategy.

Du et al.^34^ roughness, profile deviation and roundness deviation were studied on the lathe. It achieved high prediction accuracy with artificial neural network based machine learning application. Researchers used random forests (RF) machine learning to estimate the dimensional accuracy and surface quality of holes^35^. Additionally recurrent neural networks methods for instance long short term memory and transformer networks, which represent the latest technology in natural language processing fields for instance speech recognition and machine translation^29^.

There are also different studies on deep learning in the literature. Researchers studied the analysis of milled surfaces using an experimental and deep learning model. They revealed that the proposed CNN model has a sensitive and thin structure that replaces high-cost Ra measuring devices^36^. Pan et al.^37^ used ultrasonic vibratory cutting technology for precision machining of W-tungsten alloy through deep learning method. More than 10% prediction accuracy has been achieved. Researchers worked on the model for machine speed prediction with deep learning. They worked on the model that included convolutional neural networks LSTM encoder-decoder architecture^38^. Wang et al. examined the advantages of deep learning for the prediction of product quality in welding processes. In addition, they focused on the deep learning technique, conventional neural networks (CNNs) and recurrent neural networks (RNNs), which are suitable for image processing and sequential modeling^39^.

The extreme learning machine has become a structure used in applications for instance 3D shape analysis and classification today. A single hidden layer feedforward neural network with N-hidden nodes is defined as in Eq. 1^40^. Here, *a*_*i*_ and *b*_*i*_ are the learning parameters. *B*_*i*_*, i*. is the weight of the hidden node. G(x) is the activation function^40,41^.

1 $$f_{N} \left( x \right) = \mathop \sum \limits_{i = 1}^{N} B_{i} , G\left( {\left( {a_{i} , b_{i} ,x} \right), x\epsilon R,{ }a_{i} { }\epsilon { }R} \right)$$

Chen et al.^41^ proposed an unsupervised attribution selection-based extreme learning machine for clustering that integrates ELM with norm editing to remove hidden neurons and cluster data directly without creating an embedding. Akusok et al.^40^ presents a new perspective on ELM solution in relation to conventional linear algebraic performance of high performance extreme learning machines for big data; and has successfully achieved the latest software and hardware performance. Zhou et al.^42^, addresses issues by proposing a new TCM method that uses only a few suitable property parameters of signals in combination with a two-layer angular core extreme learning machine. Wu et al.^43^, The article explored a voice recognition based ELM pattern detection method to end product poorness caused by cutting tool breakage or wear in the machining process.

Looking at the studies published in WEDM; The determined change of machining parameters and machining performance outputs for instance Kerf, Ra, Mrr and metallurgical structure change was analyzed by various studies. The detailed literature review showed that the number of studies on the extent to which the workpiece changes its properties when exposed to different processes is limited and the limited number of published studies are not comprehensive. Experimental and artificial intelligence-based theoretical studies on this subject will make a great contribution to this field. Therefore, this study investigated the effects of conductivity, microhardness and microstructure of the specimens with WEDM parameters, and the results were predicted for Ra, kerf, MRR and WWR outputs with deep learning and extreme learning machine.

## Material and methods

**Figure 2:**
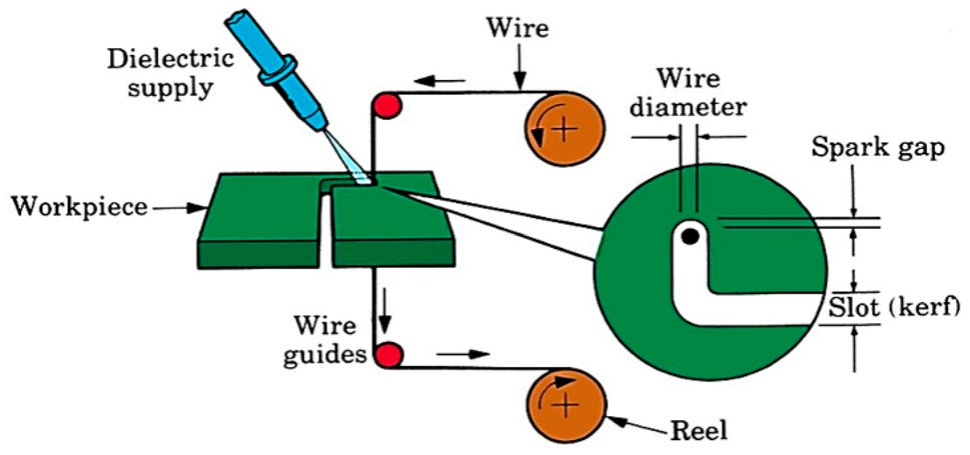
WEDM process^44^.

**Figure 3:**
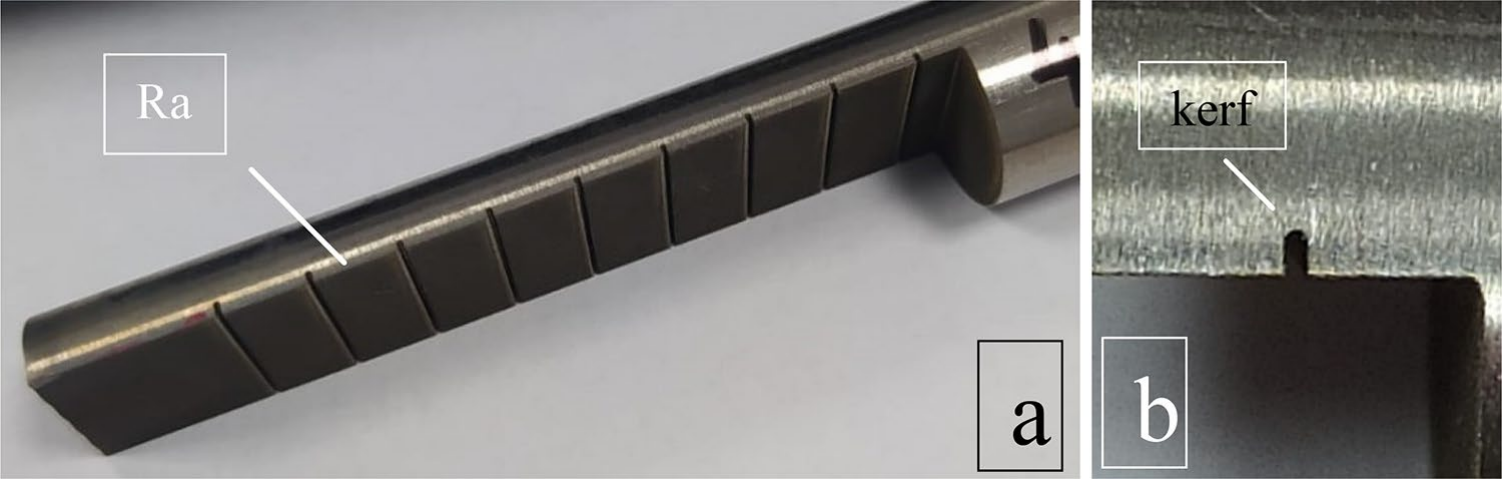
(**a**) Ra and (**b**) kerf images of this study.

In the first phase of this study, different process types were processed on the samples. The aim here is to determine the changes in properties such as microstructure, microhardness and conductivity that occur in the structure of hardox 400 steel when exposed to different processes. Then, the samples affected by these changes were processed in WEDM and measurement results (Figs. 2, 3) with the box-behnken experimental design. The results were analyzed in the Minitab 21 program. In the second phase of the study, a prediction models were created with DL and ELM for the Ra, Kerf, MRR and WWR to be made using these data. Tensorflow was used for DL and hpelm was used for ELM in the study. A linear regression models are also presented to compare with the results.

### Process type

Hardox steel was subjected to different processes (Table 1) to change its structure. These processes were carried out with five different samples as heat treatment, cold forging, plasma welding, mig-mag welding and commercial sample. In this context, it is expected that the microstructure, microhardness and electrical conductivity of Hardox steel samples will vary.

**Table 1:** Process type.

Process	Application
Master sample	Commercial sample
Heat treatment	Heating to 960 °C (15 min) holding and quenching + heating to 240 °C and holding 3 h after air cooling
Cold forging	Cold drawing will be done 3 times in a row, reducing the diameter by 2% in each process
Plasma welding	180 Amper, 0.125 mm/min feed rate
Mig-mag welding	180 Amper, 0.125 mm/min feed rate

### Master sample

Hardox is a versatile wear resistant steels with hardness of 400 HV. The chemical composition of Hardox 400 is shown in Table 2 and its mechanical properties are shown in Table 3. It is well suited for additional wear applications requiring high toughness, excellent weldability and bendability. They are also wear resistant steels in the form of versatile and wear resistant round bars and the high toughness provides good weldability. It is commercially available quenched to high tensile strength and hardness values. Hardox round bars open up new possibilities for stronger product designs. In addition, these steels help optimize workshop processes such as machining and welding.

**Table 2:** Chemical composition of Hardox 400 steel.

Element	C	Si	Mn	P	S	Cr	Ni	Mo	B
(wt% max)	0.32	0.70	1.60	0.025	0.01	1.40	1.50	0.60	0.004

**Table 3:** Mechanical properties of Hardox Steel.

Tensile strength (Mpa)	Yield strength (Mpa)	Impact energy KV (J)	Hardness (HBW)	Elongation (%)
1550	1300	30	430	8

### Heat treatment

Conductivity, microhardness and microstructures of samples of Hardox Steel exposed to heat treatments were examined in this study. The purpose of this study is to obtain a distinct change on the sample micro structures by applying heat treatment and to determine the effect of this changed microstructures on machinibility with WEDM. The heat treatment of the samples was carried out in the Protherm 442 furnace. The samples were prepared in accordance with the EN 10325 heat treatment standard. Table 1 shows the heat treatment parameters for the austenitizing and subsequent tempering of the samples. Heat treatments applied to Hardox Steel that Heating to 960 °C (15 min) holding and quenching and Heating to 240 °C and holding 3 h after air cooling.

### Cold forging

Cold drawn steel, such as cold rolled steel, is machined at room temperature. With the cold drawing process, it is ensured that the hot rolled products are brought to more precise measurement tolerances, more durable and superior surface quality is obtained. The desired hardness can be achieved on the surface without heat treatment, but since the structure of the material is interfered with, problems may occur in the internal structure and surface of the material. As a result of cold drawing, yield and tensile stress and hardness increase, while ductility decreases. In this study, cold drawing process was applied 3 times in succession by reducing the diameter of 2% in each process. The samples were drawn in accordance with the EN 10278 cold drawing standard.

### Plasma welding and mig-mag welding

Hardox steels were joined by different welding (Plasma and Mig-mag) methods, but at the same amperage and feed rate. The samples were prepared in accordance with the EN 17632 Mig-Mag welding standard. The changes of these parameters on weld zone, heat affected zone width, microstructure, microhardness, conductivity were investigated. The effects of these changes on machinability in WEDM were investigated. Ra, Kerf, MRR and WWR measurements in welded samples include the average of the measurements of the weld zone and the heat affected zone.

### Design of experiment for WEDM

RSM shows an experimental setup that aims to obtain the highest number of dependent variables on the response surface with the least possible number of observable values. Experimental design is made to examine the relations of the variables with the objective or response functions. However, during the experimental design, one variable is changed at a time, as in the classical approach. However, this approach is difficult and time consuming, especially in multivariate systems. On the other hand, the statistical design of experiments, reduces the number of experiments to be performed, takes into account the interactions between variables, and can be used for optimization of operating parameters in multivariate systems^45^. In this study, Box–Behnken statistical experimental design was used to investigate the effects of six independent variables on response functions and to determine the conditions that maximize Ra, Kerf, MRR, WWR efficiency. The Box–Behnken statistical experiment design method offers an empirical relationship between the response function and the independent variables. The approximation is a first-order model if it gives a good result on the response surface of the system as a linear function of the independent variable Eq. 2^45^;

2 $$y = \beta_{0} + \beta_{1} x_{1} + \beta_{2 } x_{2} + \ldots + \beta_{k} \beta_{k} + \varepsilon$$

If the response surface of the system has a curvature, a quadratic model may be more appropriate Eq. 3^1,45^;

3 $$y = \beta_{0} \mathop \sum \limits_{i = 1}^{n} \beta_{i} X_{i} + \mathop \sum \limits_{i = 0}^{n} \beta_{ii} X_{i}^{2} + \mathop \sum \limits_{i = 0}^{n} \mathop \sum \limits_{j = 1}^{n} \beta_{ij} X_{i} X_{j} + \varepsilon_{0}$$

As a result of structure change, the machinability of WEDM was examined. Machinability parameters were determined according to the box-behnken surface response methodology. Parameters and their levels are shown in Table 4. The result of Ra, Kerf, MRR, WWR were analyzed and graphics were examined by using Minitab 21 program. Experimental studies were performed on an ONA AF25 precision CNC WEDM. The following were used in the experimental setup; Ø 0.25 mm brass wire was used as the electrode and the dimensions of hardox 400 samples were Ø 40 mm in all experiments. Experiments were carried out to determine the variability in input parameters and the cutting width, surface quality and material removal rates on the workpiece.

**Table 4:** Parameters and levels of Box behnken surface response methodology.

Continuous Factors	Level values				
	Low	High			
Time off (µs)	200	300			
Current (A)	4	6			
Dielectric (bar)	4	16			
Wire feed (m/min)	4	10			
Wire tension (g)	12	20			

Categorical factor	1	2	3	4	5
Process type	Master sample	Heat treated	Cold forged	Plasma welded	Mig-mag welded

### Optimization with artificial intelligence

Optimization is a mathematical discipline and an approach to determining the optimific in a quantitatively well-defined sense. The math optimization of processes controlled by differential equations has shown significant advances in recent years. This has been applied to a large spectrum of disciplines for instance mathematics, engineering, economics. Optimization theory covers algorithms for solving optimization problems and their analysis. An optimization problem specifies an objective function to be maximized or minimized with constraints.

The prediction models were created with DL and ELM for the modeling studies to be made using these data. Before the analysis, the independent variables were normalized between [0,1], and the dependent variables were not normalized. Python 3.9 was used in the study. The normalization process was applied for all three methods (DL, ELM and regression). Rmsprop and adam methods as optimization algorithms were tried. sigmoid, relu, tanh and linear as activation functions were applied. In the experiments, the number of hidden layers was determined as 1, 2 and 3. The number of neurons in each hidden layer will vary from 6 to 150. 90% of the data were determined for training and 10% for testing (207 were used as training data and 23 were used as test data). Epochs were determined as 1000 (Table 5). Linear regression models is also introduced to compare with the efficiency of the results.

**Table 5:** DL and ELM parameters.

Parameters	DL	Basic ELM	P-ELM	OP-ELM
Optimization algorithm	Adam,RmsPROP	–	–	–
Normalization	Min–Max scaling	Min–Max scaling	Min–Max scaling	Min–Max scaling
Input layer activation function	ReLu, sigmoid	Sigmoid	Sigmoid	Sigmoid
Output layer activation function	–	Linear	Linear	Linear
Number of input layer neuron	6	6	6	6
Number of output layer neuron	1	6	6	6
Number of hidden layers	3	1	1	1
Number of hidden#1 layer neuron	6, 12	10, 12, 15	80, 120, 150	80, 120, 150
Number of hidden#2 layer neuron	6, 12	–	–	–
Number of hidden#3 layer neuron	6, 12	–	–	–
Learning rate	0.001			
Batch size	16			
Training size	0.9	0.9	0.9	0.9
Test size	0.1	0.1	0.1	0.1
Epochs	1000	–	–	–

## Results and discussion

### Response surface metodology

The Ra, Kerf, MRR, WWR values obtained from the box behnken design of the parameters and subsequent 230 experiments in WEDM are shown in Table 6. Experiment results were analyzed using Minitab 21 program and graphs were drawn. After the samples were subjected to different processes, the changing hardness and microstructures also had an effect on the machinability.

**Table 6:** WEDM parameters and results.

Exp. no	Toff	Current	Dielectric	Wf	Wt	Sample	Ra	Kerf	MRR	WWR
1	250	6	16	7	16	Mig-mag welded	2.57	271	0.267	0.133
2	300	5	10	7	12	Mig-mag welded	3.64	384	0.378	0.188
3	250	5	4	4	16	Heat treated	3.22	315	0.310	0.154
4	300	6	10	7	16	Plasma welded	3.21	339	0.333	0.165
5	300	5	4	7	16	Plasma welded	3.63	383	0.377	0.187
6	250	6	10	10	16	Heat treated	1.95	215	0.211	0.105
7	300	4	10	7	16	Mig-Mag welded	3.62	382	0.376	0.187
⋮	⋮	⋮	⋮	⋮	⋮	⋮	⋮	⋮	⋮	⋮
222	250	5	10	7	16	Cold forged	2.64	280	0.276	0.137
223	250	5	10	7	16	Cold forged	2.62	270	0.266	0.132
224	250	6	10	4	16	Master sample	2.95	311	0.307	0.152
225	250	4	16	7	16	Mig-mag welded	3.08	325	0.320	0.159
226	250	5	10	10	20	Plasma welded	2.6	274	0.270	0.134
227	250	6	10	7	12	Heat treated	2.55	269	0.265	0.132
228	200	5	10	10	16	Heat treated	2.15	205	0.209	0.104
229	250	6	10	7	20	Plasma welded	2.59	273	0.269	0.134
230	250	4	10	4	16	Plasma welded	3.33	359	0.354	0.176

**Figure 4:**
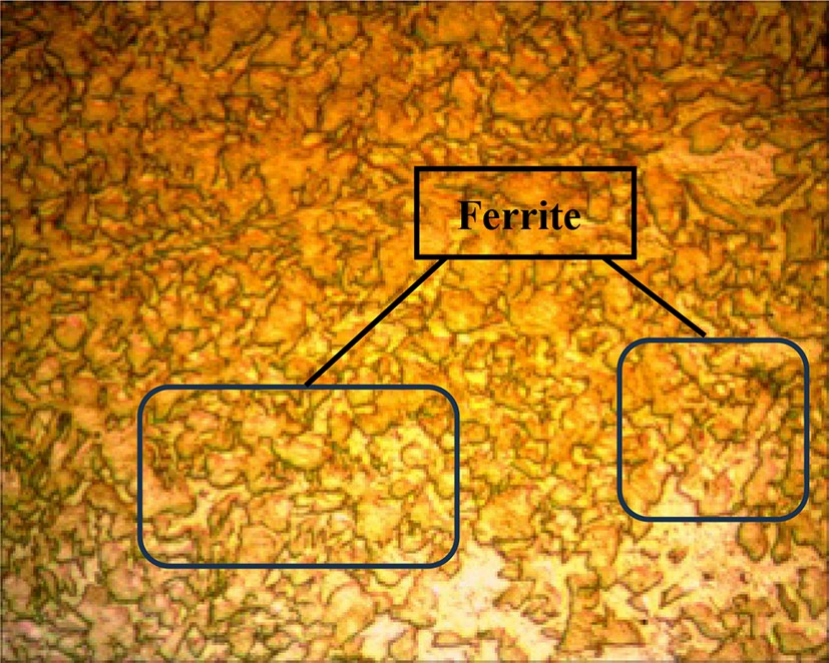
Master sample Hardox microstructures.

**Figure 5:**
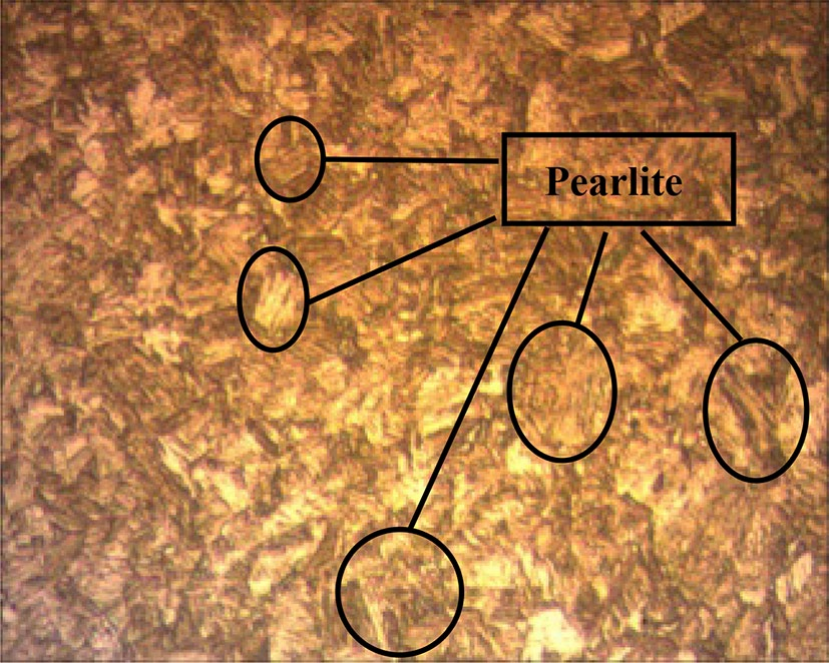
Heat treated Hardox microstructures.

After the heat treatment, the α-ferrite phase volume increased in the sample, while the pearlite phase volume decreased^2^. Observable decreases were detected in Ra and kerf values due to the relative decrease in hardness. In Figs. 4 and 5 microstructures of tempered samples at commercial and Heating to 960 °C holding and quenching + Heating to 240 °C and holding 3 h after air cooling are given respectively. In these microstructures as the tempering temperature increased α-ferrite phase volume also increased whereas pearlite phase volume decreased. Similar results were also encountered in literatüre^3,46^.

### Microhardness

**Figure 6:**
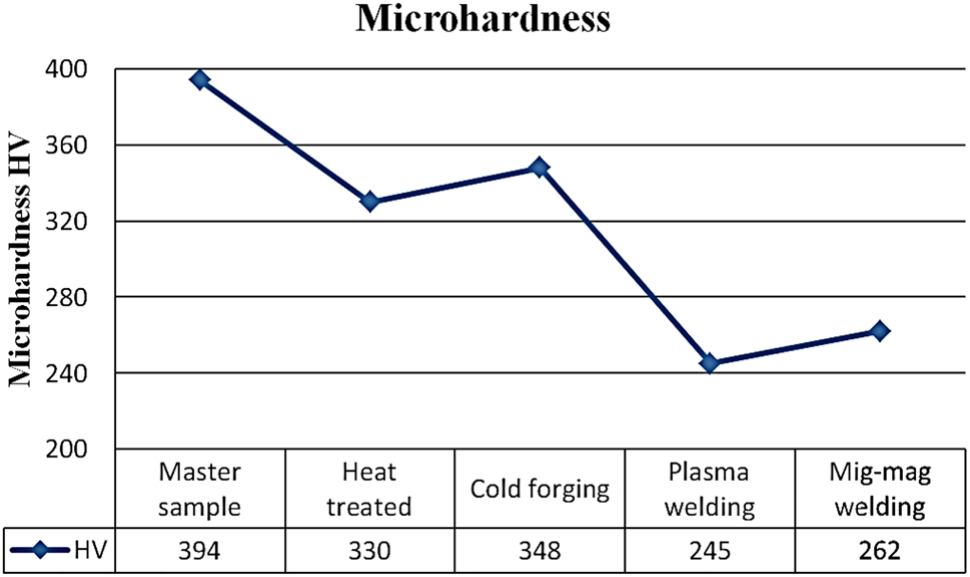
Microhardness result.

The hardness values of the samples were measured and given in Fig. 6. According to the hardness results, the commercial sample hardness was 394. Following the heat treatment, the hardness of the sample was measured as 330. The sample hardness was 348 by cold drawing process. The microhardness values of the welded samples were relatively lower than the other processes. It was measured as plasma welded 245 and mig-mag welded 262. Microhardness also affected machinability due to changes in microstructure and conductivity. Master sample has tempered martensite structure (Fig. 2). These hardox steels are produced thoroughly hardened and presented as such^2^. Similar results were also encountered in literatüre^2,46^.

In hardox bars, 2% diameter reduction was achieved in each cold drawing process. After this process repeated twice, a relative increase in hardness values was expected. This affected its machinability and caused Ra, kerf values to be lower than the commercial sample. The underlying reason for giving similar results with heat treatment can be explained by increased conductivity values.

**Figure 7:**
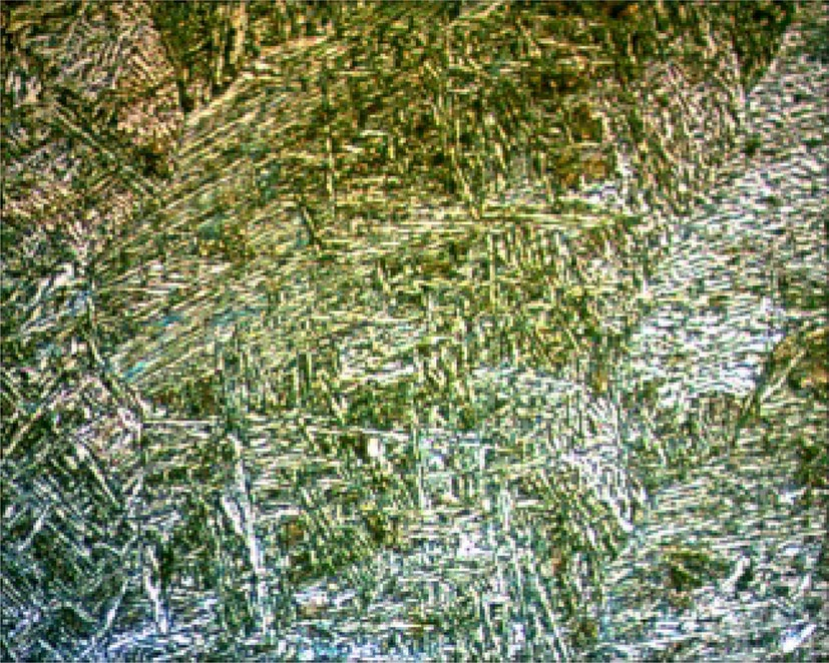
Plasma welded Hardox microstructures.

The effect of heat input and subsequent cooling process to the sample on the microstructure is very important. Since the welding heat of Hardox rods will create a tempering effect in this region after welding^3^, it is inevitable that a fine perlite structure will form in the microstructure. In addition, heat input affected the electrical conductivity in welded samples. As a result, Ra affected the Kerf, MRR and WWR outputs. Lamel bainite in the weld metal zone microstructure of the sample significantly affected the machinability (Fig. 7).

**Figure 8:**
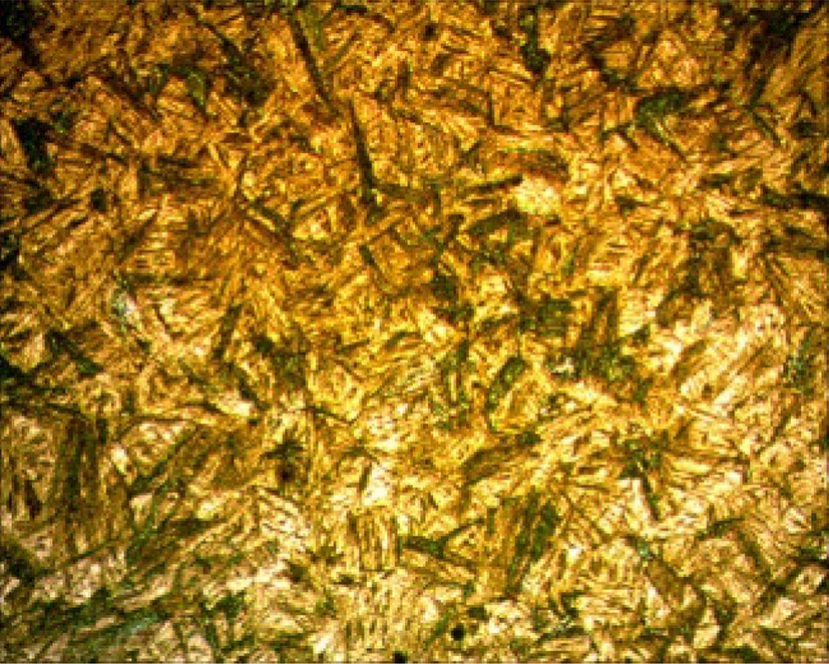
Mig-mag welded Hardox microstructures.

In Fig. 8, the weld zone microstructure of the sample welded using 180 A with the MAG method is given. Here, the α-ferrite phase morphology is dendritic. In addition, a thin pearlite phase was observed between the dendritic phases.

Analysis of variance (ANOVA) tests were also performed for the responses and the results are presented in Tables 7, 10, 13 and 16. As seen in Table 7, the model F value was calculated as 86.04 according to the Ra ANOVA. In addition, since the model *p* value is < 0.05, it shows that the determined variables and the model are statistically significant. In addition, all WEDM parameters are extremely important for the model according to F and *P* values. According to the ANOVA results, the most important parameter affecting the surface roughness was found to be the hardox samples exposed to different processes (40.40%).

**Table 7:** Analysis of variance for Ra.

Source	DF	Seq SS	Contribution (%)	Adj SS	Adj MS	F-value	*P* value
Model	44	37.6023	95.34	37.6023	0.85460	86.04	0.000
Linear	9	37.1177	94.11	37.1177	4.12419	415.22	0.000
Toff	1	4.0365	10.23	4.0365	4.03651	406.39	0.000
Current	1	3.9206	9.94	3.9206	3.92055	394.72	0.000
Dielctric	1	4.1314	10.48	4.1314	4.13141	415.94	0.000
Wf	1	4.6803	11.87	4.6803	4.68028	471.20	0.000
Wt	1	4.4133	11.19	4.4133	4.41330	444.33	0.000
Sample	4	15.9356	40.40	15.9356	3.98391	401.10	0.000
Square	5	0.0325	0.08	0.0325	0.00650	0.65	0.659
Toff * toff	1	0.0083	0.02	0.0034	0.00344	0.35	0.557
Current * current	1	0.0240	0.06	0.0214	0.02136	2.15	0.144
Dielctric * dielctric	1	0.0000	0.00	0.0000	0.00004	0.00	0.949
Wf * Wf	1	0.0001	0.00	0.0000	0.00001	0.00	0.971
Wt + Wt	1	0.0002	0.00	0.0002	0.00020	0.02	0.888
2-Way ınteraction	30	0.4522	1.15	0.4522	0.01507	1.52	0.051
Toff * current	1	0.0004	0.00	0.0004	0.00040	0.04	0.840
Toff * dielctric	1	0.0135	0.03	0.0135	0.01352	1.36	0.245
Toff * Wf	1	0.0198	0.05	0.0198	0.01985	2.00	0.159
Toff * Wt	1	0.0022	0.01	0.0022	0.00221	0.22	0.638
Toff * sample	4	0.0586	0.15	0.0586	0.01465	1.47	0.212
Current * dielctric	1	0.0192	0.05	0.0192	0.01922	1.94	0.166
Current * Wf	1	0.0650	0.16	0.0650	0.06498	6.54	0.011
Current * Wt	1	0.0020	0.01	0.0020	0.00200	0.20	0.654
Current * sample	4	0.0396	0.10	0.0396	0.00991	1.00	0.410
Dielctric * Wf	1	0.0003	0.00	0.0003	0.00032	0.03	0.858
Dielctric * Wt	1	0.0016	0.00	0.0016	0.00162	0.16	0.687
Dielctric * sample	4	0.0423	0.11	0.0423	0.01058	1.07	0.375
Wf * Wt	1	0.0320	0.08	0.0320	0.03200	3.22	0.074
Wf * sample	4	0.0641	0.16	0.0641	0.01602	1.61	0.173
Wt * sample	4	0.0914	0.23	0.0914	0.02285	2.30	0.060
Error	185	1.8375	4.66	1.8375	0.00993		
Lack-of-fit	160	1.4871	3.77	1.4871	0.00929	0.66	0.932
Pure error	25	0.3504	0.89	0.3504	0.01402		
Total	229	39.4399	100.00				

In the analysis for Ra, Model R^2^ value was obtained as 0.9534. Estimated and adjust R^2^ values were calculated as 0.9290 and 0.9423, and these two values show a statistically significant agreement (Table 8).

**Table 8:** Model summary for Ra.

S	R-sq	R-sq (adj)	R-sq (pred)
0.0996624	95.34%	94.23%	92.90%

Regression analyses are performed for the modeling and analysis of different variables with a relationship between one dependent variable and one or more independent variables^22^. Linear regression models are relatively simple and provide an mathematical formula that can produce predictions. In this study, the equations for estimation of the Ra, kerf, MRR and WWR were calculated using regression analysis. Response function equations with determined coefficients for Ra, Kerf, MRR, WWR efficiency are shown in Tables 9, 12, 15 and 18. Signs and magnitudes of the coefficients in the response functions show the effect of the independent variables on the response function and its importance in this context. The most ideal regression equations for ra are given in Table 9, depending on the results of the Box behnken experimental design.

**Table 9:** Regression equations for Ra.

Sample		
Master sample	Ra	3.63 + 0.00681 Toff − 0.247 Current + 0.0138 Dielctric + 0.1343 Wf- 0.0476 Wt − 0.000004 Toff * Toff + 0.0221 Current * Current + 0.000027 Dielctric * Dielctric − 0.00006 Wf * Wf + 0.000133 Wt * Wt + 0.000090 Toff * Current − 0.000087 Toff * Dielctric − 0.000210 Toff * Wf + 0.000053 Toff * Wt − 0.00517 Current * Dielctric − 0.01900 Current * Wf − 0.00250 Current * Wt − 0.00022 Dielctric * Wf − 0.000375 Dielctric * Wt − 0.00333 Wf * Wt
Heat treated	Ra	2.84 + 0.00687 Toff − 0.230 Current + 0.0140 Dielctric + 0.1120 Wf − 0.0244 Wt − 0.000004 Toff * Toff + 0.0221 Current * Current + 0.000027 Dielctric * Dielctric − 0.00006 Wf * Wf + 0.000133 Wt * Wt + 0.000090 Toff * Current − 0.000087 Toff * Dielctric − 0.000210 Toff * Wf + 0.000053 Toff * Wt − 0.00517 Current * Dielctric − 0.01900 Current * Wf − 0.00250 Current * Wt − 0.00022 Dielctric * Wf − 0.000375 Dielctric * Wt − 0.00333 Wf * Wt
Plasma welded	Ra	3.39 + 0.00700 Toff − 0.222 Current + 0.0229 Dielctric + 0.1258 Wf − 0.0337 Wt − 0.000004 Toff * Toff + 0.0221 Current * Current + 0.000027 Dielctric * Dielctric − 0.00006 Wf * Wf + 0.000133 Wt * Wt + 0.000090 Toff * Current − 0.000087 Toff * Dielctric − 0.000210 Toff * Wf + 0.000053 Toff * Wt − 0.00517 Current * Dielctric − 0.01900 Current * Wf- 0.00250 Current * Wt − 0.00022 Dielctric * Wf − 0.000375 Dielctric * Wt − 0.00333 Wf * Wt
Mig-mag welded	Ra	3.20 + 0.00771 Toff − 0.222 Current + 0.0194 Dielctric + 0.1318 Wf − 0.0325 Wt − 0.000004 Toff * Toff + 0.0221 Current * Current + 0.000027 Dielctric * Dielctric − 0.00006 Wf * Wf + 0.000133 Wt * Wt + 0.000090 Toff * Current − 0.000087 Toff * Dielctric − 0.000210 Toff * Wf + 0.000053 Toff * Wt − 0.00517 Current * Dielctric − 0.01900 Current * Wf − 0.00250 Current * Wt − 0.00022 Dielctric * Wf − 0.000375 Dielctric * Wt − 0.00333 Wf * Wt
Cold forged	Ra	3.31 + 0.00818 Toff − 0.281 Current + 0.0132 Dielctric + 0.1124 Wf − 0.0444 Wt − 0.000004 Toff * Toff + 0.0221 Current * Current + 0.000027 Dielctric * Dielctric − 0.00006 Wf * Wf + 0.000133 Wt * Wt + 0.000090 Toff * Current − 0.000087 Toff * Dielctric − 0.000210 Toff * Wf + 0.000053 Toff * Wt − 0.00517 Current * Dielctric − 0.01900 Current * Wf − 0.00250 Current * Wt − 0.00022 Dielctric * Wf − 0.000375 Dielctric * Wt − 0.00333 Wf * Wt

**Figure 9:**
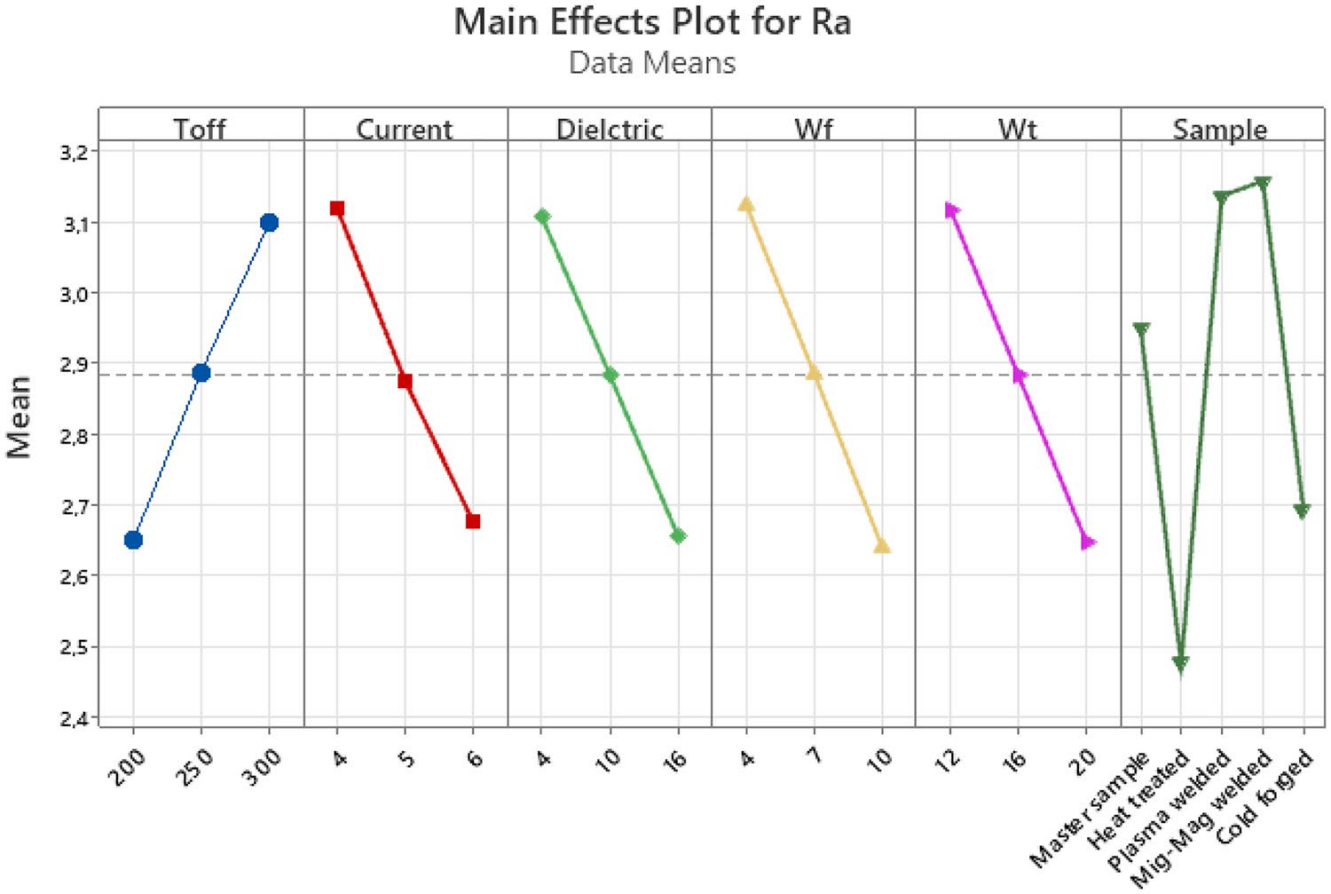
Main effect plot for Ra.

When Fig. 9 is examined, an increase in Ra values was observed with the increase of Toff, one of the WEDM parameters. A general decrease in Ra values was observed with the increase of Current, Dielectric, wire feed and wire tension values. When the effects of the samples on Ra were examined according to the process type, the lowest Ra values were acquired in the heat-treated, cold forged, master sample, plasma welded and mig-mag welded processes, respectively. Similar results were also encountered in literatüre^1,12,28^.

As seen in Table 10, the model F value was calculated as 90.21 according to the kerf ANOVA. In addition, since the model *p* value is < 0.05, it shows that the determined variables and the model are statistically significant. In addition, all WEDM parameters were found to be extremely important for the model according to F and *P* (< 0.05) values. According to the ANOVA results, the most important parameter affecting the kerf was found to be the hardox samples exposed to different processes (41.92%).

**Table 10:** Analysis of variance for kerf.

Source	DF	Seq SS	Contribution (%)	Adj SS	Adj MS	F-value	*P* value
Model	44	415,102	95.55	415,102	9434.1	90.21	0.000
Linear	9	409,690	94.30	409,690	45,521.1	435.28	0.000
Toff	1	46,868	10.79	46,868	46,868.3	448.16	0.000
Current	1	42,576	9.80	42,576	42,576.1	407.12	0.000
Dielctric	1	44,111	10.15	44,111	44,110.8	421.79	0.000
Wf	1	45,840	10.55	45,840	45,839.8	438.32	0.000
Wt	1	48,172	11.09	48,172	48,171.8	460.62	0.000
Sample	4	182,124	41.92	182,124	45,530.9	435.37	0.000
Square	5	507	0.12	507	101.3	0.97	0.438
Toff * toff	1	69	0.02	19	18.7	0.18	0.673
Current * current	1	427	0.10	381	381.3	3.65	0.058
Dielctric * dielctric	1	3	0.00	1	0.8	0.01	0.929
Wf * Wf	1	7	0.00	8	7.7	0.07	0.787
Wt * Wt	1	0	0.00	0	0.2	0.00	0.961
2-Way Interaction	30	4905	1.13	4905	163.5	1.56	0.040
Toff * current	1	5	0.00	5	5.2	0.05	0.824
Toff * dielctric	1	169	0.04	169	168.8	1.61	0.205
Toff * Wf	1	0	0.00	0	0.2	0.00	0.963
Toff * Wt	1	46	0.01	46	45.6	0.44	0.510
Toff * sample	4	488	0.11	488	122.0	1.17	0.327
Current * dielctric	1	214	0.05	214	213.9	2.05	0.154
Current * Wf	1	648	0.15	648	648.1	6.20	0.014
Current * Wt	1	9	0.00	9	9.4	0.09	0.765
Current * sample	4	526	0.12	526	131.4	1.26	0.289
Dielctric * Wf	1	45	0.01	45	45.2	0.43	0.512
Dielctric * Wt	1	10	0.00	10	10.5	0.10	0.752
Dielctric * sample	4	495	0.11	495	123.9	1.18	0.319
Wf * Wt	1	266	0.06	266	265.9	2.54	0.113
Wf * sample	4	1020	0.23	1020	255.1	2.44	0.049
Wt * sample	4	962	0.22	962	240.5	2.30	0.060
Error	185	19,347	4.45	19,347	104.6		
Lack-of-fit	160	15,353	3.53	15,353	96.0	0.60	0.968
Pure error	25	3994	0.92	3994	159.8		
Total	229	434,449	100.00				

In the analysis for Kerf, the Model R^2^ value was obtained as 0.9555. Estimated and adjust R^2^ values were calculated as 0.9324 and 0.9449, and these two values show a statistically significant agreement (Table 11). The most ideal regression equations for kerf are given in Table 12, depending on the results of the Box behnken experimental design.

**Table 11:** Model summary for kerf.

S	R-sq	R-sq (adj)	R-sq (pred)
10.2264	95.55%	94.49%	93.24%

**Table 12:** Regression equations for kerf.

Sample		
Master sample	Kerf	438 + 0.582 Toff − 29.9 Current + 1.96 Dielctric + 7.34 Wf − 6.04 Wt − 0.000262 Toff * Toff + 2.96 Current * Current − 0.0038 Dielctric * Dielctric + 0.047 Wf * Wf + 0.0047 Wt * Wt − 0.0102 Toff * Current − 0.00968 Toff * Dielctric + 0.0007 Toff * Wf + 0.0075 Toff * Wt − 0.545 Current * Dielctric − 1.897 Current * Wf − 0.171 Current * Wt − 0.083 Dielctric * Wf − 0.0302 Dielctric * Wt − 0.304 Wf * Wt
Heat treated	Kerf	339 + 0.599 Toff − 26.1 Current + 2.40 Dielctric + 5.05 Wf − 3.79 Wt − 0.000262 Toff * Toff + 2.96 Current * Current − 0.0038 Dielctric * Dielctric + 0.047 Wf * Wf + 0.0047 Wt * Wt − 0.0102 Toff * Current − 0.00968 Toff * Dielctric + 0.0007 Toff * Wf + 0.0075 Toff * Wt − 0.545 Current * Dielctric − 1.897 Current * Wf − 0.171 Current * Wt − 0.083 Dielctric * Wf − 0.0302 Dielctric * Wt − 0.304 Wf * Wt
Plasma welded	Kerf	401 + 0.622 Toff − 27.9 Current + 3.05 Dielctric + 7.28 Wf − 4.43 Wt − 0.000262 Toff * Toff + 2.96 Current * Current − 0.0038 Dielctric * Dielctric + 0.047 Wf * Wf + 0.0047 Wt * Wt − 0.0102 Toff * Current − 0.00968 Toff * Dielctric + 0.0007 Toff * Wf + 0.0075 Toff * Wt − 0.545 Current * Dielctric − 1.897 Current * Wf − 0.171 Current * Wt − 0.083 Dielctric * Wf − 0.0302 Dielctric * Wt − 0.304 Wf * Wt
Mig-mag welded	Kerf	387 + 0.665 Toff − 26.2 Current + 2.54 Dielctric + 7.41 Wf − 4.40 Wt − 0.000262 Toff * Toff + 2.96 Current * Current − 0.0038 Dielctric * Dielctric + 0.047 Wf * Wf + 0.0047 Wt * Wt − 0.0102 Toff * Current − 0.00968 Toff * Dielctric + 0.0007 Toff * Wf + 0.0075 Toff * Wt − 0.545 Current * Dielctric − 1.897 Current * Wf − 0.171 Current * Wt − 0.083 Dielctric * Wf − 0.0302 Dielctric * Wt − 0.304 Wf * Wt
Cold forged	Kerf	404 + 0.719 Toff − 32.9 Current + 1.93 Dielctric + 4.80 Wf − 5.77 Wt − 0.000262 Toff * Toff + 2.96 Current * Current − 0.0038 Dielctric * Dielctric + 0.047 Wf * Wf + 0.0047 Wt * Wt − 0.0102 Toff * Current − 0.00968 Toff * Dielctric + 0.0007 Toff * Wf + 0.0075 Toff * Wt − 0.545 Current * Dielctric − 1.897 Current * Wf − 0.171 Current * Wt − 0.083 Dielctric * Wf − 0.0302 Dielctric * Wt − 0.304 Wf * Wt

**Figure 10:**
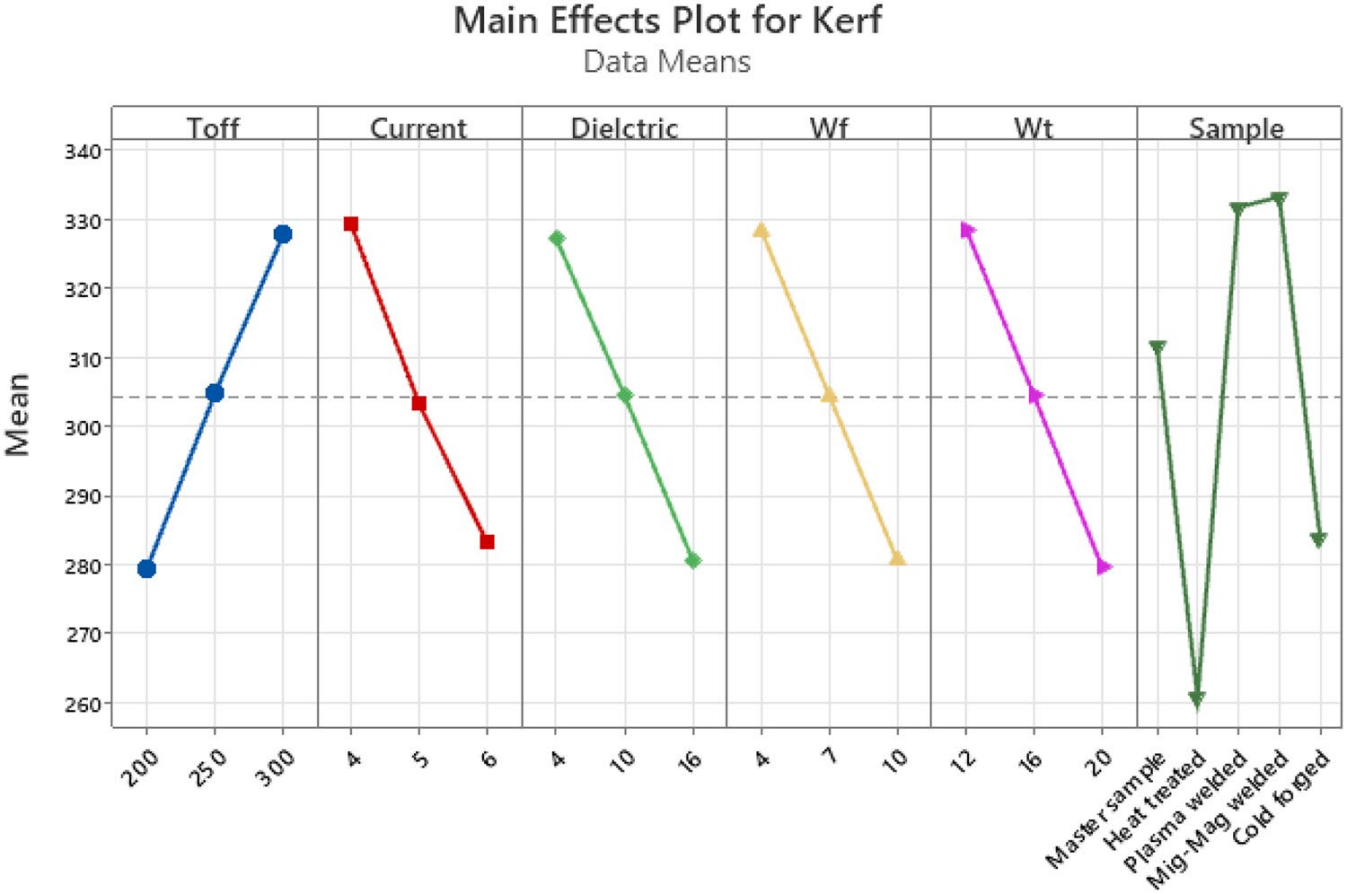
Main effect plot for kerf.

When Fig. 10 is examined, an increase in kerf values was observed with the increase of Toff, one of the WEDM parameters. A general decrease in kerf values was observed with the increase in Current, Dielectric, wire feed and wire tension values. When the effects of the samples on the kerf were examined according to the process type, the lowest kerf values were obtained in the heat-treated, cold forged, master sample, plasma welded and mig-mag welded processes, respectively. There are similar results in some studies in literature^2,47–51^.

As seen in Table 13, the model F value was calculated as 92.11, according to the MRR ANOVA. In addition, since the model *p* value is < 0.05, it shows that the determined variables and the model are statistically significant. In addition, all WEDM parameters were found to be extremely important for the model according to F and *P* (< 0.05) values. According to the ANOVA results, the most important parameter affecting the MRR was found to be the hardox samples exposed to different processes (41.87%).

**Table 13:** Analysis of variance for MRR.

Source	DF	Seq SS	Contribution (%)	Adj SS	Adj MS	F-value	*P* value
Model	44	0.401223	95.63	0.401223	0.009119	92.11	0.000
Linear	9	0.396049	94.40	0.396049	0.044005	444.54	0.000
Toff	1	0.044966	10.72	0.044966	0.044966	454.25	0.000
Current	1	0.042077	10.03	0.042077	0.042077	425.06	0.000
Dielctric	1	0.042758	10.19	0.042758	0.042758	431.94	0.000
Wf	1	0.043562	10.38	0.043562	0.043562	440.06	0.000
Wt	1	0.047008	11.20	0.047008	0.047008	474.87	0.000
Sample	4	0.175677	41.67	0.175677	0.043919	443.67	0.000
Square	5	0.000491	0.12	0.000491	0.000098	0.99	0.424
Toff * toff	1	0.000073	0.02	0.000018	0.000018	0.18	0.672
Current * current	1	0.000396	0.09	0.000369	0.000369	3.73	0.055
Dielctric * dielctric	1	0.000006	0.00	0.000001	0.000001	0.01	0.907
Wf * Wf	1	0.000013	0.00	0.000015	0.000015	0.16	0.693
Wt * Wt	1	0.000003	0.00	0.000003	0.000003	0.03	0.854
2-Way ınteraction	30	0.004683	1.12	0.004683	0.000156	1.58	0.037
Toff * current	1	0.000006	0.00	0.000006	0.000006	0.06	0.811
Toff * dielctric	1	0.000174	0.04	0.000174	0.000174	1.76	0.187
Toff * Wf	1	0.000003	0.00	0.000003	0.000003	0.03	0.858
Toff * Wt	1	0.000051	0.01	0.000051	0.000051	0.51	0.475
Toff * sample	4	0.000470	0.11	0.000470	0.000117	1.19	0.318
Current * dielctric	1	0.000232	0.06	0.000232	0.000232	2.35	0.127
Current * Wf	1	0.000638	0.15	0.000638	0.000638	6.45	0.012
Current * Wt	1	0.000000	0.00	0.000000	0.000000	0.00	0.959
Current * sample	4	0.000468	0.11	0.000468	0.000117	1.18	0.321
Dielctric * Wf	1	0.000046	0.01	0.000046	0.000046	0.46	0.497
Dielctric * Wt	1	0.000012	0.00	0.000012	0.000012	0.12	0.728
Dielctric * sample	4	0.000451	0.11	0.000451	0.000113	1.14	0.340
Wf * Wt	1	0.000249	0.06	0.000249	0.000249	2.52	0.114
Wf * sample	4	0.000903	0.22	0.000903	0.000226	2.28	0.062
Wt * sample	4	0.000981	0.23	0.000981	0.000245	2.48	0.046
Error	185	0.018313	4.37	0.018313	0.000099		
Lack-of-fit	160	0.014439	3.44	0.014439	0.000090	0.58	0.975
Pure error	25	0.003874	0.92	0.003874	0.000155		
Total	229	0.419536	100.00				

In the analysis for MRR, the Model R^2^ value was obtained as 0.9563. Estimated and adjust R^2^ values were calculated as 0.9339 and 0.9460, and these two values show a statistically significant agreement (Table 14). The most ideal regression equations for mrr are given in Table 15, depending on the results of the Box behnken experimental design.

**Table 14:** Model summary for Mrr.

S	R-sq	R-sq (adj)	R-sq (pred)
0.0099494	95.63%	94.60%	93.39%

**Table 15:** Regression equations for Mrr.

Sample		
Master sample	MRR	0.440 + 0.000592 Toff − 0.0314 Current + 0.00227 Dielctric + 0.00779 Wf- 0.00720 Wt − 0.000000 Toff * Toff + 0.00291 Current * Current − 0.000005 Dielctric * Dielctric + 0.000066 Wf * Wf + 0.000017 Wt * Wt − 0.000011 Toff * Current − 0.000010 Toff * Dielctric − 0.000003 Toff * Wf + 0.000008 Toff * Wt − 0.000568 Current * Dielctric − 0.001883 Current * Wf − 0.000029 Current * Wt − 0.000084 Dielctric * Wf − 0.000032 Dielctric * Wt − 0.000294 Wf * Wt
Heat treated	MRR	0.340 + 0.000604 Toff − 0.0274 Current + 0.00267 Dielctric + 0.00579 Wf − 0.00483 Wt − 0.000000 Toff * Toff + 0.00291 Current * Current − 0.000005 Dielctric * Dielctric + 0.000066 Wf * Wf + 0.000017 Wt * Wt − 0.000011 Toff * Current − 0.000010 Toff * Dielctric − 0.000003 Toff * Wf + 0.000008 Toff * Wt − 0.000568 Current * Dielctric − 0.001883 Current * Wf − 0.000029 Current * Wt − 0.000084 Dielctric * Wf − 0.000032 Dielctric * Wt − 0.000294 Wf * Wt
Plasma welded	MRR	0.404 + 0.000631 Toff − 0.0292 Current + 0.00329 Dielctric + 0.00783 Wf − 0.00565 Wt − 0.000000 Toff * Toff + 0.00291 Current * Current − 0.000005 Dielctric * Dielctric + 0.000066 Wf * Wf + 0.000017 Wt * Wt − 0.000011 Toff * Current − 0.000010 Toff * Dielctric − 0.000003 Toff * Wf + 0.000008 Toff * Wt − 0.000568 Current * Dielctric − 0.001883 Current * Wf − 0.000029 Current * Wt − 0.000084 Dielctric * Wf − 0.000032 Dielctric * Wt − 0.000294 Wf * Wt
Mig-Mag welded	MRR	0.397 + 0.000672 Toff − 0.0282 Current + 0.00281 Dielctric + 0.00786 Wf − 0.00579 Wt − 0.000000 Toff * Toff + 0.00291 Current * Current − 0.000005 Dielctric * Dielctric + 0.000066 Wf * Wf + 0.000017 Wt * Wt − 0.000011 Toff * Current − 0.000010 Toff * Dielctric − 0.000003 Toff * Wf + 0.000008 Toff * Wt − 0.000568 Current * Dielctric − 0.001883 Current * Wf − 0.000029 Current * Wt − 0.000084 Dielctric * Wf − 0.000032 Dielctric * Wt − 0.000294 Wf * Wt
Cold forged	MRR	0.407 + 0.000725 Toff − 0.0341 Current + 0.00220 Dielctric + 0.00534 Wf − 0.00694 Wt − 0.000000 Toff * Toff + 0.00291 Current * Current − 0.000005 Dielctric * Dielctric + 0.000066 Wf * Wf + 0.000017 Wt * Wt − 0.000011 Toff * Current − 0.000010 Toff * Dielctric − 0.000003 Toff * Wf + 0.000008 Toff * Wt − 0.000568 Current * Dielctric − 0.001883 Current * Wf − 0.000029 Current * Wt − 0.000084 Dielctric * Wf − 0.000032 Dielctric * Wt − 0.000294 Wf * Wt

**Figure 11:**
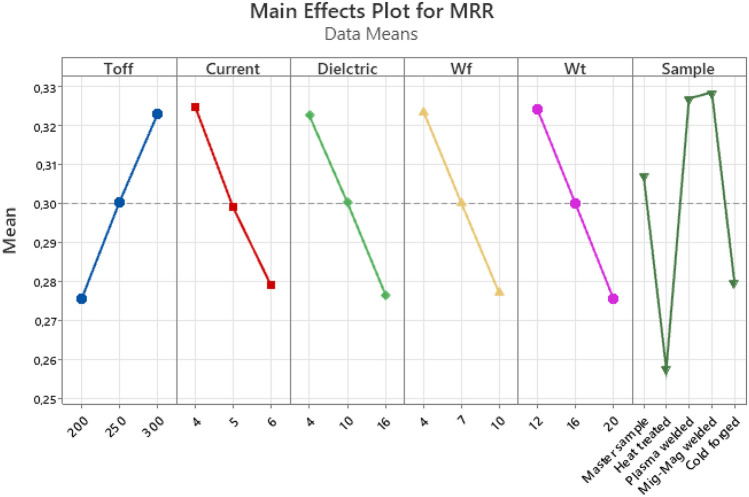
Main effect plot for MRR.

When Fig. 11 is examined, an increase in MRR values was observed with the increase of Toff, one of the WEDM parameters. Contrary to Ra and Kerf, it is desirable to have high MRR values. A general decrease in kerf values was observed with the increase in Current, Dielectric, wire feed and wire tension values. When the effects of the samples on the MRR were examined according to the process type, the highest MRR values were obtained in the mig-mag welded, plasma welded, cold forged, master sample and heat-treated processes, respectively. There are similar results in some studies in literatüre^2,6,40,52,53^.

As seen in Table 16, the model F value was calculated as 92.12, according to the wwr ANOVA. In addition, since the model *p* value is < 0.05, it shows that the determined variables and the model are statistically significant. In addition, all WEDM parameters were found to be extremely important for the model according to F and *P* (< 0.05) values. According to the ANOVA results, the most important parameter affecting the WWR was found to be the hardox samples exposed to different processes (41.88%).

**Table 16:** Analysis of variance for WWR.

Source	DF	Seq SS	Contribution (%)	Adj SS	Adj MS	F-value	*P* value
Model	44	0.099077	95.63	0.099077	0.002252	92.12	0.000
Linear	9	0.097799	94.40	0.097799	0.010867	444.54	0.000
Toff	1	0.011104	10.72	0.011104	0.011104	454.25	0.000
Current	1	0.010390	10.03	0.010390	0.010390	425.06	0.000
Dielctric	1	0.010559	10.19	0.010559	0.010559	431.94	0.000
Wf	1	0.010757	10.38	0.010757	0.010757	440.06	0.000
Wt	1	0.011608	11.20	0.011608	0.011608	474.87	0.000
Sample	4	0.043381	41.88	0.043381	0.010845	443.67	0.000
Square	5	0.000121	0.12	0.000121	0.000024	0.99	0.424
Toff * toff	1	0.000018	0.02	0.000004	0.000004	0.18	0.672
Current * current	1	0.000098	0.09	0.000091	0.000091	3.73	0.055
Dielctric * dielctric	1	0.000002	0.00	0.000000	0.000000	0.01	0.907
Wf * Wf	1	0.000003	0.00	0.000004	0.000004	0.16	0.693
Wt * Wt	1	0.000001	0.00	0.000001	0.000001	0.03	0.854
2-Way ınteraction	30	0.001156	1.12	0.001156	0.000039	1.58	0.037
Toff * current	1	0.000001	0.00	0.000001	0.000001	0.06	0.811
Toff * dielctric	1	0.000043	0.04	0.000043	0.000043	1.76	0.187
Toff * Wf	1	0.000001	0.00	0.000001	0.000001	0.03	0.858
Toff * Wt	1	0.000013	0.01	0.000013	0.000013	0.51	0.475
Toff * sample	4	0.000116	0.11	0.000116	0.000029	1.19	0.318
Current * dielctric	1	0.000057	0.06	0.000057	0.000057	2.35	0.127
Current * Wf	1	0.000158	0.15	0.000158	0.000158	6.45	0.012
Current * Wt	1	0.000000	0.00	0.000000	0.000000	0.00	0.959
Current * sample	4	0.000115	0.11	0.000115	0.000029	1.18	0.321
Dielctric * Wf	1	0.000011	0.01	0.000011	0.000011	0.46	0.497
Dielctric * Wt	1	0.000003	0.00	0.000003	0.000003	0.12	0.728
Dielctric * sample	4	0.000111	0.11	0.000111	0.000028	1.14	0.340
Wf * Wt	1	0.000062	0.06	0.000062	0.000062	2.52	0.114
Wf * sample	4	0.000223	0.22	0.000223	0.000056	2.28	0.062
Wt * sample	4	0.000242	0.23	0.000242	0.000061	2.48	0.046
Error	185	0.004522	4.37	0.004522	0.000024		
Lack-of-fit	160	0.003566	3.44	0.003566	0.000022	0.58	0.975
Pure error	25	0.000957	0.92	0.000957	0.000038		
Total	229	0.103599	100.00				

In the analysis for WWR, the Model R^2^ value was obtained as 0.9561. Estimated and adjust R^2^ values were calculated as 0.9341 and 0.9462, and these two values show a statistically significant agreement (Table 17). The most ideal regression equations for wwr are given in Table 18, depending on the results of the Box behnken experimental design.

**Table 17:** Model summary for WWR.

S	R-sq	R-sq (adj)	R-sq (pred)
0.0049441	95.61%	94.62%	93.41%

**Table 18:** Regression equations for WWR.

Sample		
Master sample	WWR	0.2188 + 0.000294 Toff − 0.0156 Current + 0.00113 Dielctric + 0.00387 Wf − 0.00358 Wt − 0.000000 Toff * Toff + 0.001446 Current * Current − 0.000002 Dielctric * Dielctric + 0.000033 Wf * Wf + 0.000009 Wt * Wt− 0.000005 Toff * Current − 0.000005 Toff * Dielctric − 0.000001 Toff * Wf + 0.000004 Toff * Wt- 0.000282 Current * Dielctric − 0.000936 Current * Wf- 0.000014 Current * Wt − 0.000042 Dielctric * Wf − 0.000016 Dielctric * Wt − 0.000146 Wf * Wt
Heat treated	WWR	0.1688 + 0.000300 Toff − 0.0136 Current + 0.00133 Dielctric + 0.00288 Wf − 0.00240 Wt − 0.000000 Toff * Toff + 0.001446 Current * Current − 0.000002 Dielctric * Dielctric + 0.000033 Wf * Wf + 0.000009 Wt * Wt − 0.000005 Toff * Current- 0.000005 Toff * Dielctric − 0.000001 Toff * Wf + 0.000004 Toff * Wt − 0.000282 Current * Dielctric − 0.000936 Current * Wf − 0.000014 Current * Wt − 0.000042 Dielctric * Wf − 0.000016 Dielctric * Wt − 0.000146 Wf * Wt
Plasma welded	WWR	0.2008 + 0.000314 Toff − 0.0145 Current + 0.00164 Dielctric + 0.00389 Wf − 0.00281 Wt − 0.000000 Toff * Toff + 0.001446 Current * Current − 0.000002 Dielctric * Dielctric + 0.000033 Wf * Wf + 0.000009 Wt * Wt − 0.000005 Toff * Current − 0.000005 Toff * Dielctric − 0.000001 Toff * Wf + 0.000004 Toff * Wt − 0.000282 Current * Dielctric − 0.000936 Current * Wf − 0.000014 Current * Wt − 0.000042 Dielctric * Wf − 0.000016 Dielctric * Wt − 0.000146 Wf * Wt
Mig-Mag welded	WWR	0.1975 + 0.000334 Toff − 0.0140 Current + 0.00140 Dielctric + 0.00391 Wf − 0.00288 Wt − 0.000000 Toff * Toff + 0.001446 Current * Current − 0.000002 Dielctric * Dielctric + 0.000033 Wf * Wf + 0.000009 Wt * Wt − 0.000005 Toff * Current − 0.000005 Toff * Dielctric − 0.000001 Toff * Wf + 0.000004 Toff * Wt- 0.000282 Current * Dielctric − 0.000936 Current * Wf − 0.000014 Current * Wt − 0.000042 Dielctric * Wf − 0.000016 Dielctric * Wt − 0.000146 Wf * Wt
Cold forged	WWR	0.2022 + 0.000360 Toff − 0.0169 Current + 0.00110 Dielctric + 0.00265 Wf − 0.00345 Wt − 0.000000 Toff * Toff + 0.001446 Current * Current − 0.000002 Dielctric * Dielctric + 0.000033 Wf * Wf + 0.000009 Wt * Wt − 0.000005 Toff * Current − 0.000005 Toff * Dielctric − 0.000001 Toff * Wf + 0.000004 Toff * Wt − 0.000282 Current * Dielctric − 0.000936 Current * Wf − 0.000014 Current * Wt − 0.000042 Dielctric * Wf − 0.000016 Dielctric * Wt − 0.000146 Wf * Wt

**Figure 12:**
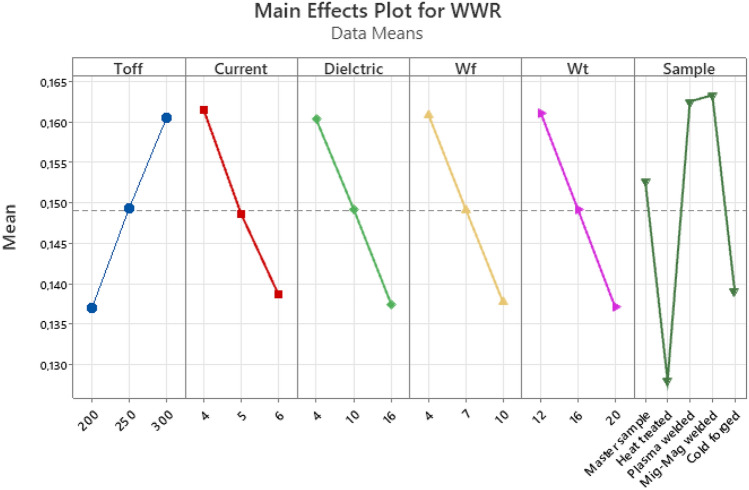
Main effect plot for WWR.

When Fig. 12 is examined, an increase in WWR values was noticed with the increase of Toff, one of the WEDM parameters. A general decrease in WWR values was observed with the increase in Current, Dielectric, wire feed and wire tension values. When the effects of the samples on the wwr were examined according to the process type, the lowest WWR values were obtained in the heat-treated, cold forged, master sample, plasma welded and mig-mag welded processes, respectively.

### Optimization with artificial intelligence

**Figure 13:**
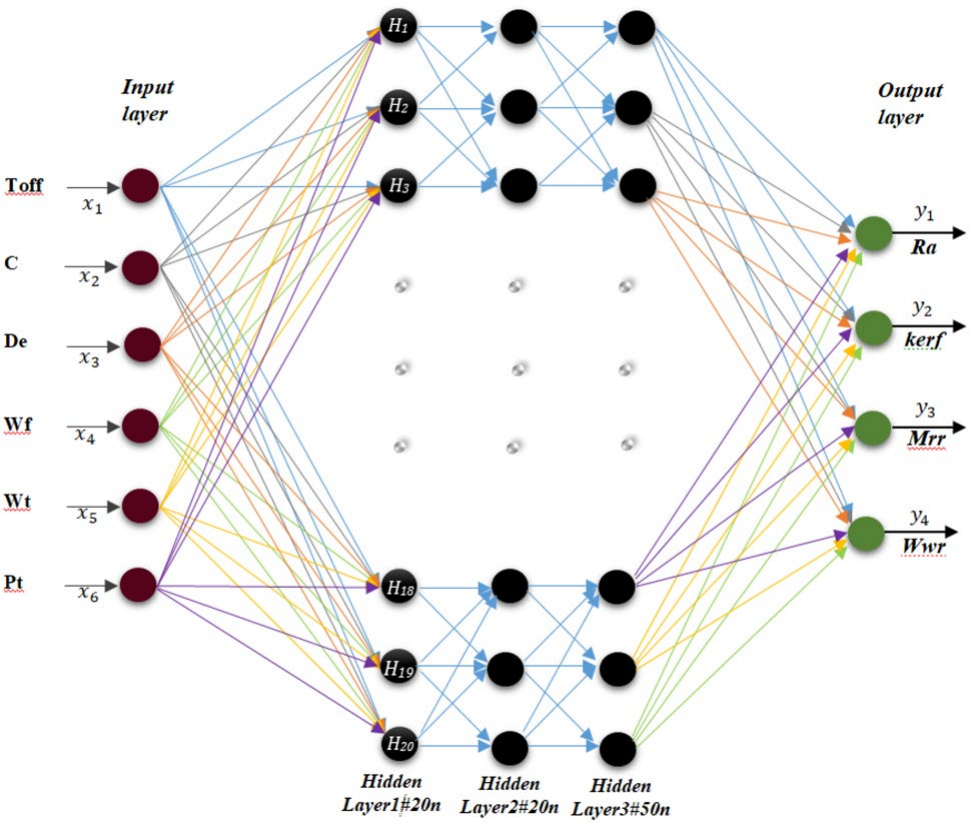
DL architecture of this study.

**Figure 14:**
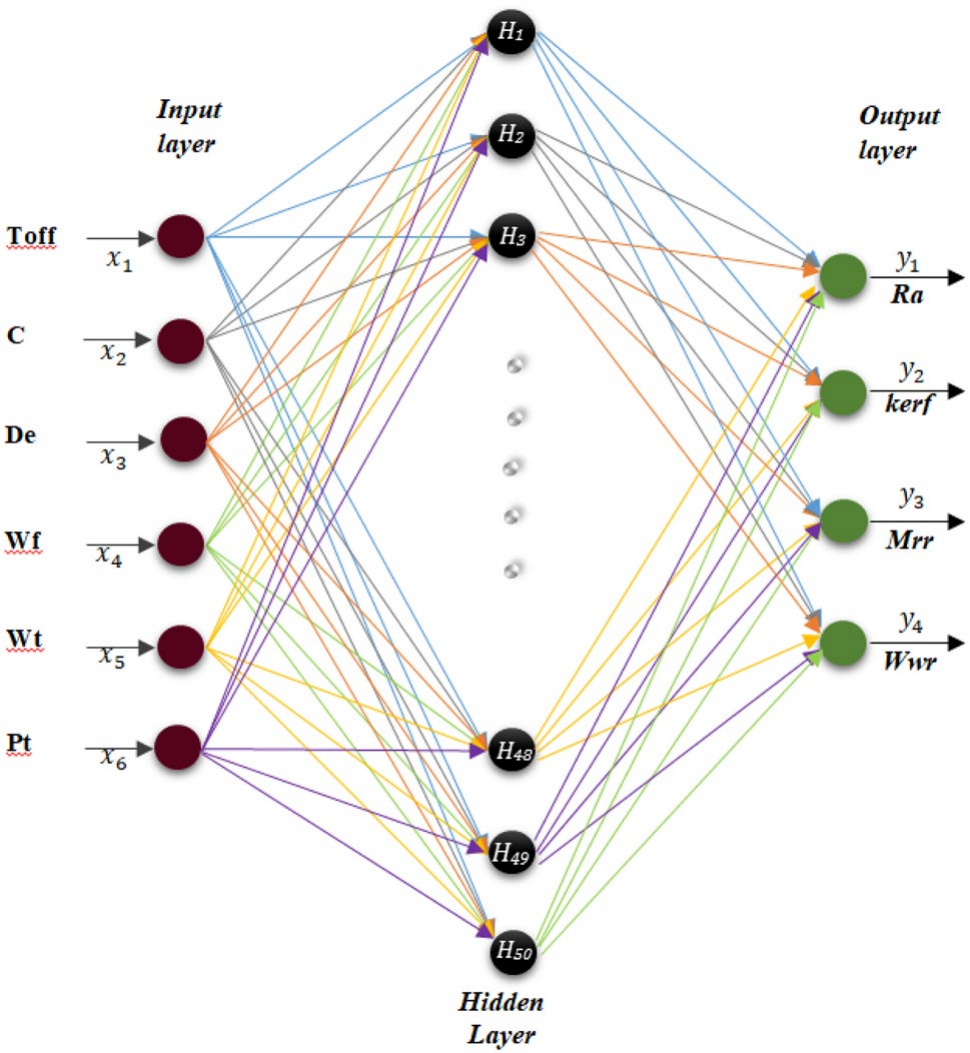
ELM architecture of this study.

In this study, 36 different trial run were applied with these DL parameters. Additionally 8 different trial run were applied with these ELM parameters. The DL and ELM model used in the study are shown in Figs. 13 and 14. The dataset for DL and ELM are set to (230 * 4). In deep learning, the maximal vigorous results were determined according to the mean square error. Adam as the optimization algorithm, sigmoid as the activation function, number of hidden layers 3, number of neurons 6 and were determined as 10% of the data to be tested. In the extreme learning machine, the activation function is sigmoid in the hidden layers and linear in the output layers. The number of neurons in both the input layers is 6 and the number of neurons in the output layers is 6.

The results are optimized with the DL and ELM. MSE values and r square values of Ra, Kerf, MRR and WWR values as a result of DL and ELM optimization are given in Table 19. It is also optimized by linear regression to compare the optimization results. In addition, the regression equations of the outputs are shown in Eqs. 4–7.

**Table 19:** Result of DL, ELM and linear regression.

	Method	Optimization algorithms (ANN)-ELM methods	Neuron numbers	Activation functions	Training MSE	Test MSE	r^2^
Ra	Linear regression				0.0295	0.0373	0.7752
	Deep learning	Adam	12, 12, 12	ReLu, ReLu, ReLu	0.0088	**0.0120**	0.9274
		RmsProp	12, 6, 6	ReLu, ReLu, ReLu	0.0094	0.0163	0.9018
	ELM	Basic ELM	10	Sigmoid	0.0295	0.0353	0.7870
		P-ELM	100	Sigmoid	0.0333	0.031	0.8130
		OP-ELM	120	Sigmoid	0.0109	0.022	0.8671
Kerf	Linear regression				328.32	405.45	0.7837
	Deep learning	Adam	6, 6, 6	Sigmoid, Sigmoid, Sigmoid	314.32	362.40	0.8067
		RmsProp	12, 6, 12	Sigmoid, Sigmoid, Sigmoid	317.17	359.55	0.8082
	ELM	Basic ELM	15	Sigmoid	287.69	360.07	0.8079
		P-ELM	80	Sigmoid	294.58	386.90	0.7937
		OP-ELM	120	Sigmoid	116.55	**248.28**	0.8676
MRR	Linear regression				0.000313	0.000395	0.7832
	Deep learning	Adam	12, 6, 6	ReLu, ReLu, ReLu	0.000069	**0.000101**	0.9444
		RmsProp	12, 6, 6	ReLu, ReLu, ReLu	0.000069	0.000179	0.9018
	ELM	Basic ELM	15	Sigmoid	0.000295	0.00038	0.7916
		P-ELM	80	Sigmoid	0.00033	0.000396	0.7831
		OP-ELM	120	Sigmoid	0.000096	0.000166	0.9091
WWR	Linear regression	Linear regression			0.000077	0.000097	0.7844
	Deep learning	Adam	6, 12, 6	ReLu, ReLu, ReLu	0.000016	**0.000037**	0.918473
		RmsProp	12, 12, 12	ReLu, ReLu, ReLu	0.000020	0.000058	0.8714
	ELM	Basic ELM	12	Sigmoid	0.000075	0.000095	0.7890
		P-ELM	80	Sigmoid	0.000082	0.000091	0.7971
		OP-ELM	120	Sigmoid	0.000028	0.000073	0.8382

Deep learning model runs for two learning algorithms (RmsProp, Adam), two activation functions (ReLU, Sigmoid) and different neuron numbers. ELM models runs for basic-ELM, P-ELM and OP-ELM. For Ra, Kerf, MRR and WWR, the best MSE value for test data are given in bold. The results are given supplementary documents.

The best test MSE value for Ra was obtained as 0.012 in DL and the r squared value 0.9274. The best test MSE value for kerf was obtained as 248.28 in ELM and r squared value 0.8676. The best MSE value for MRR was obtained as 0.000101 in DL and the r squared value 0.944. The best MSE value for WWR was obtained as 0.000037 in DL and the r squared value 0.918473. As a result, it was concluded that different optimization methods can be applied according to different outputs (Ra, Kerf, MRR, WWR). It also shows that artificial intelligence-based optimization methods give successful estimation results about Ra, Kerf, MRR, WWR values.

**Figure 15:**
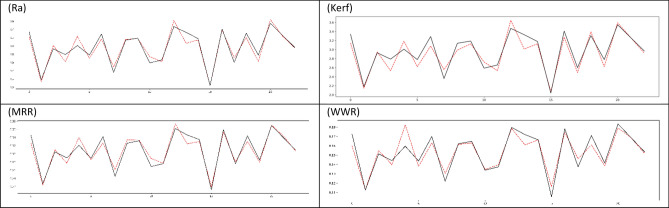
Original data and model output comparison graph for best test MSE Value.

Comparative graphs of the actual value in the test values of the best model obtained for Ra, Kerf, MRR and WWR and the predicted values of the model are given in the Fig. 15. In the graphs, the black and solid lines show the actual values, and the red and dashed lines show the predicted values of the best model. The graphs show that the best model achieves results very close to the true values.

The coefficients of the regression equations obtained without normalization on the data are given Eqs. 4–7.

4 $$y_{Ra} = \, 0.454 + 0.0041*Toff - 0.2214*Current - 0.0378*Dielectric - 0.085*Wire \, feed - 0.058*Wire \, tension + 0.1053*Process \, type$$

5 $$y_{kerf} = \, 469.28 + 0.0044*Toff - 22.97*Current - 3.87*Dielectric - 8.44*Wire \, feed - 6.131*Wire \, tension + 11.168*Process \, type$$

6 $$y_{mrr} = \, 0.4634 + 0.00043*Toff - 0.0227*Current - 0.0038*Dielectric - 0.0082*Wire \, feed - 0.0060*Wire \, tension + 0.0110*Process \, type$$

7 $$y_{wwr} = \, 0.231 + 0.00021*Toff - 0.011*Current - 0.0019*Dielectric - 0.0040*Wire \, feed - 0.00303*Wire \, tension + 0.0054*Process \, type$$

## Conclusion

Hardox steel was subjected to different processes to change its structure. These processes were carried out with five different samples as heat treatment, cold forging, plasma welding, mig-mag welding and commercial sample. In this context, the microstructure, microhardness and electrical conductivity of Hardox steel samples are expected to vary. Then, the samples affected by these changes were processed in WEDM with the box-behnken experimental design. Ra, Kerf, MRR and WWR results were analyzed in Minitab 21 program.

In the next phase of the study, a prediction model was created for Ra, Kerf, MRR and WWR with DL and ELM using these data. Anaconda Python 3.9 version was used as a program in the optimization study. Additionally, a linear regression models are presented to compare the results. According to these results, ideal DL and ELM models have been presented for future studies.

According to the experimental results;

- The lowest Ra values were obtained in heat-treated, cold forged, master sample, plasma welded and mig-mag welded processes, respectively. The best Ra (surface roughness) value of 1.92 µm was obtained in the heat treated sample and in the experiment with a time off of 250 µs.
- Model F value in ANOVA analysis for Ra is 86.04. In addition, the model showed that the determined variables and the model were statistically significant since the *p* value was < 0.05.
- WEDM parameters are extremely important for Ra compared to F and *P* values.
- In the analysis made for Ra, the model r^2^ value was obtained as 0.9534.
- The lowest kerf values were obtained in heat-treated, cold forged, master sample, plasma welded and mig-mag welded processes, respectively. The best kerf value of 200 µ was obtained in the heat treated sample and in the experiment with a time off of 200 µs.
- In the ANOVA analysis for Kerf, the model F value is 90.21. In addition, the model showed that the determined variables and the model were statistically significant since the *p* value was < 0.05.
- WEDM parameters are extremely important for kerf compared to F and *P* values.
- In the analysis made for Kerf, the model r^2^ value was obtained as 0.9555.
- Contrary to Ra and kerf, it is desirable to have high mrr values. On average, the highest mrr values were obtained in mig-mag welded, plasma welded, cold forged, master sample and heat-treated processes, respectively. The best mrr value of 200 g min^−1^ was obtained in the mig-mag welded sample and in the experiment with a time off of 300 µs.
- Model F value in ANOVA analysis for Mrr is 92.12. In addition, the model showed that the determined variables and the model were statistically significant since the *p* value was < 0.05.
- WEDM parameters are extremely important for mrr compared to F and *P* values.
- In the analysis made for Mrr, the model r^2^ value was obtained as 0.9563.
- The lowest wwr values were obtained in heat-treated, cold forged, master sample, plasma welded and mig-mag welded processes, respectively. The best wwr value of 0.098 g was obtained in the heat treated sample and in the experiment with a time off of 200 µs.
- Model F value in ANOVA analysis for Wwr is 92.12. In addition, the model showed that the determined variables and the model were statistically significant since the *p* value was < 0.05.
- WEDM parameters are extremely important for wwr compared to F and *P* values.
- In the analysis made for WWR, the model r^2^ value was obtained as 0.09561.

In the analysis made with artificial intelligence systems;

- The best test MSE value for Ra was obtained as 0.012 in DL and the r squared value 0.9274.
- The best test MSE value for Kerf was obtained as 248.28 in ELM and r squared value 0.8676.
- The best MSE value for MRR was obtained as 0.000101 in DL and the r squared value 0.9444.
- The best MSE value for WWR was obtained as 0.000037 in DL and the r squared value 0.9184

As a result, it was concluded that different optimization methods can be applied according to different outputs (Ra, Kerf, MRR, WWR). It also shows that artificial intelligence-based optimization methods give successful estimation results about Ra, Kerf, MRR, WWR values. According to these results, ideal DL and ELM models have been presented for future studies.

## Data Availability

All the raw data of analysis are available as supplementary data. Any other data generated or analyzed during this study are available from the corresponding authors on reasonable request. https://docs.google.com/spreadsheets/d/1BbrfVpeJvSvvjXV5HjVqyY9Fbl8gTu4l/edit?usp=sharing&ouid=104439447976128858155&rtpof=true&sd=true.

## Abbreviations

AI: Artificial intelligence
DL: Deep learning
ML: Machine learning
ANN: Artificial neural networks
ELM: Extreme learning machine
P-ELM: Pruned-ELM
OP-ELM: Optimum pruned-ELM
WEDM: Wire electrical discharge machining
Ra: Surface roughness
RSM: Response surface methodology
X-RD: X-ray diffraction
MSE: Mean squared error
MRR: Material removal rate
WWR: Wire wear rate
GRA: Grey relational analysis
SEM: Scanning electron microscope
HTS: Heat treated sample
IPC: Ignition pulse current
TBTP: Time between two pulses
SV: Servo reference voltage
De: Dielectric
WF: Wire feed
WT: Wire tension
σ: Conductivity
P: Resistivity
